# Attribute-Based Verifiable Conditional Proxy Re-Encryption Scheme

**DOI:** 10.3390/e25050822

**Published:** 2023-05-19

**Authors:** Yongli Tang, Minglu Jin, Hui Meng, Li Yang, Chengfu Zheng

**Affiliations:** College of Software, Henan Polytechnic University, Jiaozuo 454000, China; yltang@hpu.edu.cn (Y.T.);

**Keywords:** proxy re-encryption, homomorphic signature, learning with errors, re-encryption verifiable

## Abstract

There are mostly semi-honest agents in cloud computing, so agents may perform unreliable calculations during the actual execution process. In this paper, an attribute-based verifiable conditional proxy re-encryption (AB-VCPRE) scheme using a homomorphic signature is proposed to solve the problem that the current attribute-based conditional proxy re-encryption (AB-CPRE) algorithm cannot detect the illegal behavior of the agent. The scheme implements robustness, that is the re-encryption ciphertext, can be verified by the verification server, showing that the received ciphertext is correctly converted by the agent from the original ciphertext, thus, meaning that illegal activities of agents can be effectively detected. In addition, the article demonstrates the reliability of the constructed AB-VCPRE scheme validation in the standard model, and proves that the scheme satisfies CPA security in the selective security model based on the learning with errors (LWE) assumption.

## 1. Introduction

As a new resource sharing in the field of information, cloud computing is constantly changing people’s lives. As an important technology in cloud computing, cloud storage is used to organize a series of different types of network storage devices to facilitate data sharing. To ensure the confidentiality of data, before being uploaded to a cloud server, user data are encrypted, however, this poses difficulties in sharing data between different users. When dealing with a significant quantity of data recipients, general encryption algorithms can significantly increase the computational and communication expenses incurred by the data owner. Proxy re-encryption (PRE) effectively solves this problem.

In 1998, Blaze et al. [1] first introduced the concept of PRE at the Euromonitor Conference. PRE is a data cipher conversion in cloud computing, which ensures both user data security and flexible access and sharing of data. However, in the traditional PRE system, it is usually one delegator that corresponds to another delegator, that is, a one-to-one model; this implies that only one client’s message can be re-encrypted at a time, necessitating a large amount of communication overhead and computation expense, which is contrary to the initial aim of cloud computing customers wanting to save money. In 2007, GREEN et al. [2] simplified the public key certificate authentication process by proposing an encryption scheme based on user identity information instead of a public key. However, the encryption process is specific to particular users and requires explicit information about the recipient. In 2009, JIAN et al. [3] suggested a strategy for conditional PRE (CPRE) based on identity proxy re-encryption. By designing a conditional ciphertext conversion method, the ciphertext can only be converted when the ciphertext meets the set conditions, enabling the assignment of partial decryption rights, but it is still in the form of a one-to-one assignment between the authorizer and the authorized person, which not only severely restricts users’ ability to selectively share data with other users at a fine-grained level, but it also has the problems of high communication costs and high computational overhead when a large number of users need to access that shared data, as well as wasting a large amount of local memory space to hold a large number of decryption keys.

Being a novel cryptographic technique that differs from conventional public key cryptography, attribute-based encryption (ABE) [4] is ideally suited for resolving data confidentiality protection and access control of ciphertext problems in cloud storage applications [5]. ABE technology can provide an effective one-to-many, fine-grained ciphertext access control solution for cloud storage data security. AB-CPRE schemes have been presented that demonstrate the advantages and properties of ABE and CPRE. However, the existing AB-PRE schemes and AB-CPRE schemes are mostly based on constructs such as linear mappings or discrete logarithmic puzzles [6,7]. Due to the advent of quantum computers, the security of traditional number theory puzzles is threatened and these schemes will become insecure. To solve this problem, a lattice cipher is proposed. It is believed that lattice-based cryptography can resist quantum attacks and has high computational efficiency. Therefore, lattice-based public key cryptography schemes have attracted wide attention in recent years.

However, all the AB-CPRE schemes [8,9,10] that are currently in use are semi-trusted agents, so they may perform unreliable calculations, which bring security problems to data sharing. Most AB-CPRE efforts focus on data privacy and access control without considering re-encryption authentication, which can lead to incorrect results for users.

Therefore, it is of interest to ensure that the re-encryption ciphertext is converted correctly from the original ciphertext. In a homomorphic encryption algorithm, the user can perform some kind of secure proxy calculation with the untrusted remote server. In this process, the server cannot see any private information. The homomorphic signature algorithm supports the signature operation consistent with the message, and the generated signature does not disclose any information related to the data set, which can meet the security requirements in the cloud environment, and is very suitable for the sensor network, network coding, and other message operation scenarios to ensure information security. This paper introduces homomorphic signature techniques in AB-CPRE, provides a verification mechanism for re-encryption performed by a verification server, and proposes a verifiable PRE scheme.

Our main contributions in this article are as follows:

- An AB-VCPRE scheme based on LWE is proposed. The scheme ensures by verification that the re-encryption ciphertext is correctly converted from the encryption ciphertext;
- Fine-grained access control is implemented. In combination with fully homomorphic encryption, the delegation policy supports any polynomial-depth boolean circuit;
- Robustness is achieved. The scheme uses a validation algorithm to achieve robustness. Forged or incorrectly shared ciphertexts can be detected by validating the re-encryption ciphertext with a validation server;
- The scheme satisfies CPA security. The ciphertext in our scheme needs to be signed and verified using an unforgeable homomorphic signature. This paper demonstrates that the constructed AB-VCPRE scheme is CPA security based on a LWE problem.

The rest of the paper is organized into seven sections. In Section 2, the related studies are described. In Section 3, the relevant definitions are introduced. In Section 4 and Section 5, we state the details of the scheme and the security analysis. Section 6 presents the efficiency analysis. The last section is a summary of the paper.

## 2. Related Work

Liang et al. [7] present an AB-PRE cryptographic primitive based on the augmented decisional bilinear Diffie–Hellman (DBDH) problem combining ABE and PRE for the first time, which empowers users to authorize in an access control environment. Li et al. [11] propose a proxy re-encryption scheme for a re-splitable threshold multi-agent, which is different from the encryption scheme on the ciphertext input and output plane and the re-encryption surface, which means the noise boundary has a wider range of choices and can ensure the security of the re-encryption key. Nunez et al. [12] propose a typical threshold proxy re-encryption scheme, which is based on a DBDH assumption, vulnerable to quantum attacks. Luo et al. [13] construct a standard lattice multi-hop AB-PRE scheme, which supports circuit access, has a short key, the key size is dependent on the depth of the circuit policy, and satisfies CPA security requirements based on the LWE problem in the selection security model. However, these PRE schemes may not show sufficient flexibility and practicality when the data owner wishes to select some but not all of the data for dissemination to certain users. Weng et al. [3] proposed a CPRE scheme where only those that satisfy the conditions can be re-encrypted, but it can only be applied to simple keyword-based conditions and will be limited in practical applications. Then, Yang et al. [8] propose a ciphertext policy-based AB-CPRE scheme, which supports a fine-grained decryption delegation. The ciphertext in the scheme is related to the access policy while the re-encryption key is related to the attributes, and the ciphertext can be re-encrypted only when the access policy satisfies the attributes. Huang et al. [14] propose PRECISE, which combines AB-CPRE with IBBE to support fine-grained re-encryption conditions for IBBE ciphertexts. Yao et al. [15] combine ciphertext authorization, key update, and ciphertext evolution to propose an improved revocable, identity-based ciphertext evolution conditional proxy re-encryption scheme for secure and efficient cloud data sharing.

The universal CPRE algorithm cannot ensure the cloud server’s integrity during the re-encryption procedure, while the homomorphic signature algorithm has unforgettable security and privacy, which can effectively verify the honesty of the proxy during the re-encryption. Therefore, this paper uses a homomorphic signature algorithm to propose a PRE scheme with encryption validating on the lattice, which can effectively detect the illegal behavior of the proxy and provide a guarantee for the safe sharing of data.

## 3. Preliminaries

### 3.1. Lattice

**Definition** **1** (lattice). *The lattice is a linear combination of group* $b_{1},b_{2},\ldots ,b_{n}$*’s linearly independent vectors’* $n\left(m\geq n\right)$ *integer coefficients in m-dimensional Euclidean space* $R^{m}$*, which is defined as:*

(1) $$L\left(B\right)=\left\{\sum_{i=1}^{n}x_{i}b_{i}:x_{i}\in \mathbb{Z},i=1,\ldots ,n\right\}.$$

**Lemma** **1** ([16]). *Take integer* q≥3*,* m≥6nlogq*,* $\sigma \geq m^{2}\omega \left(\sqrt{\log m}\right)$*, there exists a* PPT *algorithm* $TrapGen\left(1^{n},1^{m},q\right)$ *that generates a matrix* $A\in {\mathbb{Z}}_{q}^{n\times m}$ *and a trapdoor* $T_{A}\in {\mathbb{Z}}^{m\times m}$ *for the lattice* $\wedge_{q}^{⊥}\left(A\right)$*,* i.e., *there is* $AT_{A}=0\mathrm{mod}q$*, such that the distribution statistics satisfied by the matrix* A *are close to a uniform distribution on* ${\mathbb{Z}}_{q}^{n\times m}$*, and* $||{\tilde{T}}_{A}||\leq O\left(\sqrt{n\log q}\right)$ *holds by an absolute margin.*

**Lemma** **2** ([17]). *Let* q>2 *and* $m>\left(n+1\right)\log q+\omega \left(\log n\right)$*. Select three uniform matrices* $D\in {\left\{-1,1\right\}}^{m\times k}$*,* $E\in {\mathbb{Z}}_{q}^{n\times m}$, *and* $F\in {\mathbb{Z}}_{q}^{n\times k}$ *at random for some polynomials with* $k=k\left(n\right)$*. Distribution* $\left(E,ED,D^{T}r\right)$ *and* $\left(E,F,D^{T}r\right)$ *are statistically indistinguishable for any vector* $r\in {\mathbb{Z}}_{q}^{m}$.

LWE is a difficult problem under lattice. Regev [18] first proposed this in 2005 and proved that the average case is just as difficult to solve for several standard cells.

**Definition** **2** (LWE). *Given positive integer* n*, integer* m≥n *and* q≥2*, choosing uniform random matrix* $A\in {\mathbb{Z}}_{q}^{n\times m}$ *and vector* $s\in {\mathbb{Z}}_{q}^{n}$*, vector* $e\leftarrow \chi^{m}$ *follows the error distribution. Given* $\left(A,A^{T}s+e\right)$*, the LWE problem is to find* s *with non-negligible probability.*

**Definition** **3** (Small integer solutions problem, SIS). *Let the defining parameters be* β*,* q *is a prime number, given positive integers* m *and* n*, select a matrix* $A\in {\mathbb{Z}}_{q}^{n\times m}$ *at random, solve for a non-zero vector of integers* $z\in {\mathbb{Z}}^{m}\backslash \left\{0\right\}$ *with* ||z||≤β*. In 1996, Ajtai presented the SIS problem in the literature [16]. The homomorphic signature used for robustness in the paper is based on the SIS problem.*

### 3.2. Related Functions and Tools

#### 3.2.1. Functions of Bits and Power2

According to the article [19], decomposing the vector into the form of an inner product can effectively control the error range of the vector. The following describes how to decompose vectors into bit representations.

For any $x\in {\mathbb{Z}}^{N}$, let $x=\sum_{i=0}^{g-1}2^{i}\cdot x^{i}\mathrm{mod}q$, $x_{i}\in {\left\{0,1\right\}}^{N}$. Output vector $Bit\left(x\right)=\left(x_{0},x_{1},\ldots ,x_{g-1}\right)\in {\left\{0,1\right\}}^{1\times Ng}$, where $g=\left\lceil \log q\right\rceil$. For any $y=\left[y_{1}|y_{2}|\ldots |y_{\ell }\right]\in {\mathbb{Z}}^{N\times \ell }$, where $y_{i}$ is a column vector, output matrix

(2) $$Power2(y)=\left[\begin{array}{cccc} y_{1} & y_{2} & \cdots & y_{\ell }\\ 2y_{1} & 2y_{2} & \cdots & 2y_{\ell }\\ \vdots & \vdots & \ddots & \vdots \\ 2^{g-1}y_{1} & 2^{g-1}y_{2} & \cdots & 2^{g-1}y_{\ell } \end{array}\right]\in {\mathbb{Z}}_{q}^{Ng\times \ell }.$$

It can be verified that for any q∈ℤ, there is $\langle Bit\left(x\right),Power2\left(y\right)\rangle =\langle x,y\rangle \in {\mathbb{Z}}_{q}^{1\times \ell }$.

#### 3.2.2. Discrete Gaussian Distribution

For integer vectors $c\in {\mathbb{Z}}^{m}$, σ>0, the discrete Gaussian distribution on the m-dimensional lattice Λ is:

(3) $$D_{\wedge ,\sigma ,c}\left(x\right)=\frac{\rho_{\sigma ,c}\left(x\right)}{\rho_{\sigma ,c}\left(\wedge \right)}=\frac{\rho_{\sigma ,c}\left(x\right)}{\sum_{x\in \wedge }\rho_{\sigma ,c}\left(x\right)},\forall x\in {\mathbb{Z}}^{m}.$$

**Lemma** **3** ([17]). *Let* q≥2*,* B *is a matrix over* ${\mathbb{Z}}_{q}^{n\times m}$ *and* m>n*. Let* $T_{B}$ *is the base of* $\wedge_{q}^{⊥}\left(B\right)$*,* $\sigma \geq ||{\tilde{T}}_{B}||\omega \left(\sqrt{\log_{2}m}\right)$*. For* $u\in {\mathbb{Z}}_{q}^{n}$*, there are:*

1. *Set the rank of* $B\in {\mathbb{Z}}_{q}^{n\times m}$ *is* n*,* $E\in {\mathbb{Z}}_{q}^{n\times m}$*,* $R\in {\left\{-1,1\right\}}^{m\times m}$*,* $\sigma \geq ||{\tilde{T}}_{B}||\omega \left(\sqrt{\log_{2}m}\right)$. *Let* $F=\left(B|BR+E\right)\in {\mathbb{Z}}_{q}^{n\times \left(2m\right)}$*, PPT algorithms* $SampleBasisLeft\left(B,BR+E,T_{B},\sigma \right)$*, where* $T_{B}$ *is the base of* $\wedge_{q}^{⊥}\left(B\right)$*, output a short base* $T_{F}\in \wedge_{q}^{⊥}\left(F\right)$ *statistical distribution to* $\psi_{\sigma }^{\left(2m\right)\times \left(2m\right)}$ *;*
2. $Sample\mathrm{Pr}e\left(B,T_{B},\sigma ,u\right)$*: There is trapdoor* $T_{B}$ *of lattice* $\wedge_{q}^{⊥}\left(B\right)$*, the real number* $\sigma \geq ||{\tilde{T}}_{B}||\cdot \omega \left(\sqrt{\log n}\right)$*, for any vector* $u\in {\mathbb{Z}}_{q}^{n}$*, a PPT algorithm* $Sample\mathrm{Pr}e\left(B,T_{B},\sigma ,u\right)$ *capable of generating a vector* e *from a distribution that is statistically close to* $D_{{\mathbb{Z}}^{m},\sigma }\left(x\right)$*, satisfying* $Be=u\left(\mathrm{mod}q\right)$*;*
3. *Let the rank of* $G\in {\mathbb{Z}}_{q}^{n\times m}$ *be* n*,* $B\in {\mathbb{Z}}_{q}^{n\times m}$*, a low-dimensional matrix* $S\in {\left\{-1,1\right\}}^{m\times m}$*, a trapdoor for the lattice* $\wedge_{q}^{⊥}\left(G\right)$*, and* $\sigma \geq ||{\tilde{T}}_{E}||\cdot ||R||\omega \left(\sqrt{\log_{2}m}\right)$*. PPT algorithm* $SampleBasisRight\left(B,G,S,T_{G},\sigma \right)$ *output a short base* $T_{\left(B|BS+G\right)}\in \wedge_{q}^{⊥}\left(B|BS+G\right)$ *with a statistical distribution close to* $\Psi_{\sigma }^{\left(2m\right)\times \left(2m\right)}$.

### 3.3. Key Homomorphism

By embedding algorithmic circuits in LWE matrices, Boneh et al. suggested an ABE approach for algorithmic circuits in their paper [20], and the method was used in many LWE-based structures, for example, predicate encryption [21], constraint PRFs [22], watermarks for PRFs [23], etc.

**Definition** **4.** *For any positive integer* k*,* d*, a* g *of* depth≤d *boolean circuit, defining families of functions* $F_{k,d}=\left\{g:{\left\{0,1\right\}}^{k}\to \left\{0,1\right\}\right\}$.

**Lemma** **4** (Fully homomorphic encryption [20,24]). *Given parameters* t, h, k, d, q, χ*, where* χ *is a B-bounded noise distribution,* h *is a security parameter,* $h\geq \left\lceil t\log q\right\rceil$*. For any matrices* $B_{1},B_{2},\ldots ,B_{\ell }\in {\mathbb{Z}}_{q}^{t\times h}$*, any boolean circuit* $g:{\left\{0,1\right\}}^{k}\to \left\{0,1\right\}$ *for any depth*≤d*,* $x\in {\left\{0,1\right\}}^{k}$*, matrix* $G\in Z_{q}^{n\times m}$*, vector* $s\in {\mathbb{Z}}_{q}^{t}$*,* $e_{i}\leftarrow \chi^{h}$ *for* $i\in \left[k\right]$*, if* $p_{i}={\left(x_{i}G+B_{i}\right)}^{T}s+e_{i},\forall i\in \left[k\right]$*,*

1. $Eval_{pk}\left(g,\left(B_{1},\ldots B_{k}\right)\right)$*: Taking a circuit* g*,* k *matrices* ${\left\{B_{i}\right\}}_{i\in \left[k\right]}$ *as input, outputs a matrix* $B_{g}$*;*
2. $Eval_{ct}\left(g,{\left\{\left(x_{i},p_{i},B_{i}\right)\right\}}_{i\in \left[k\right]}\right)$*: Given a circuit* g*,* k *matrices* ${\left\{B_{i}\right\}}_{i\in \left[k\right]}$*, a vector* $x\in {\left\{0,1\right\}}^{k}$ *and* k *vectors* $\left\{p_{1},\ldots ,p_{k}\right\}$*, outputs a vector* $p_{g}$*, satisfying* $p_{g}={\left(B_{g}+g\left(x\right)G\right)}^{T}s+e_{g}$*, where* $B_{g}=Eval_{pk}\left(g,\left\{B_{1},\ldots ,B_{k}\right\}\right)$*,* $||e_{g}||\leq B\sqrt{h}{\left(1+h\right)}^{d}$ *with all but negligible probability;*
3. $Eval_{sim}\left(g,{\left\{S_{i},x_{i}^{*}\right\}}_{i\in \left[k\right]},A\right)$*: On input a circuit* g*, a vector* $x^{*}\in {\left\{0,1\right\}}^{k}$*,* k *matrices* ${\left\{S_{i}\right\}}_{i\in [k]}$*, a matrix* $A\in {\mathbb{Z}}_{q}^{t\times h}$*, outputs a matrix* $S_{g}$ *satisfying* $AS_{g}-g\left(x^{*}\right)=B_{g}$*, where* $||S_{g}|{|}_{2}\leq 20\sqrt{h}{\left(1+h\right)}^{d}<{\left(1+h\right)}^{d+1}$ *with all but negligible probability.*

### 3.4. Homomorphic Signature

A homomorphic signature is a valid signature that permits any entity to conduct a sequence of operations on the original message and its signature without the signing private key.

**Definition** **5** (Homomorphic signature). *The probabilistic polynomial-time algorithm* $\left(KG,Sign,SignEval,Verify\right)$ *is included in the following tuple is the homomorphic signature (HS) scheme:*

1. HS.KG(p,d,N)*: Take a safety parameter* p*, a circuit depth* d, *and a message length* N *as input, output a signature private key* hssk *and a verification key* hsvk*;*
2. $HS.Sign\left(hssk,M\right)$*: Accept as inputs the message* M *requiring signature and* hssk*, output the signature* σ*;*
3. HS.SEval(g,σ)*: Take an evaluation circuit* $g:{\left\{0,1\right\}}^{N}\to \left\{0,1\right\}$ *and signature* σ *as input, output a homomorphic calculation signature* $\sigma^{*}$*;*
4. $HS.Verify(hsvk,y,g,\sigma^{*})$*: Take* hsvk*, a message* y*, a circuit* g *and a signature* $\sigma^{*}$*, the verification algorithm either accepts the signature (outputs 1) or rejects it (outputs 0).*

Correctness. On input p,d,N∈ℤ, $HS.KG(p,d,N)\to \left(hsvk,\;hssk\right)$, $M\in {\left\{0,\;1\right\}}^{N}$, HS.Sign(hssk,M)→σ, any circuit $g:{\left\{0,\;1\right\}}^{N}\to \left\{0,\;1\right\}$ with a depth d, $g\left(M\right)\to y$, the equation below holds:

(4) $$\mathrm{Pr}\left[HS.Verify\left(hsvk,y,g,HS.SEval\left(g,\sigma \right)\right)=1\right]=1.$$

### 3.5. Robustness

A key component of the AB-VCPRE design is robustness. The fundamental tenet is that by re-encryption key sharing, an adversary cannot create ciphertext that is falsely obtained yet can be correctly authenticated. The following game $Expt_{A}^{Rb}$ describes the robustness of the AB-VCPRE scheme.

During the guessing phase, the adversary outputs the appropriate ciphertext $CT^{*}$ satisfies $Verfy\left(hsvk,CT^{*}\right)=1$ while Setup, KeyGen query, ReKeyGen query, and ReEnc query interact as specified in Definition 6. 

The adversary’s advantage is characterized as $Adv_{A}^{Rb}=\mathrm{Pr}\left[Expt_{A}^{Rb}\left(\lambda \right)=1\;\right].$

## 4. The Model of AB-VCPRE with Re-Encryption Verification

### 4.1. Scheme Definition

**Figure 1:**
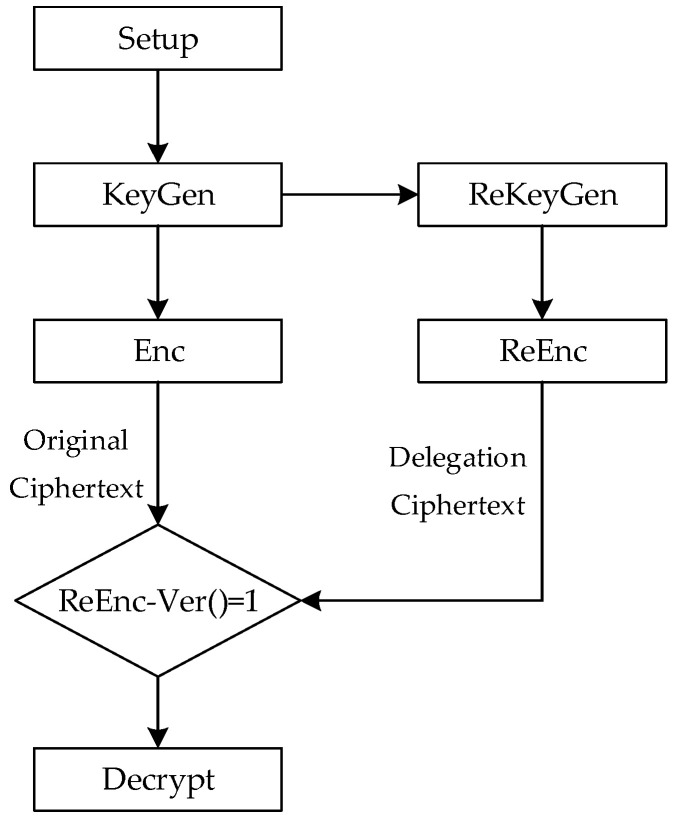
Flow chart of AB-VCPRE.

An AB-VCPRE scheme consists of seven algorithms. The specific flow chart is shown in Figure 1. In comparison to the standard AB-VCPRE, a verification method called ReEnc−Ver is added to check for an honest transformation of the ciphertext. The ReEnc−Ver algorithm is publicly verifiable because all that is required are the original ciphertext and the corresponding re-encryption ciphertext.

1. Setup(n): Input security parameter n, output public parameters pp;
2. KeyGen(pp,α): Given pp, output the public/private key pair $\left(pk_{\alpha },sk_{\alpha }\right)$ for user α;
3. $Enc\left(pp,pk_{\alpha },\mu ,x\right)$: Taking pp, $pk_{\alpha }$, plaintext μ, and an attribute vector x as input, output a related ciphertext $CT_{\alpha }$ with x;
4. $Dec(pp,sk_{\alpha },CT_{\alpha })$: Taking pp, $sk_{\alpha }$, and $CT_{\alpha }$ as input, output a message μ;
5. ReKeyGenpp,skα,pkβ,f: Input pp, $sk_{\alpha }$ of user α, $pk_{\beta }$ of user β, and a control policy/function f, returns the re-encryption key $RK_{\alpha ,f\to \beta }$ related to f and the corresponding signature, outputs the re-encryption verification key $VK_{\alpha \to \beta }$ from user α to user β;
6. ReEncpp,RKα,f→β,CTα: With pp, $pk_{\alpha }$ of user α, $CT_{\alpha }$ associated with x, and $RK_{\alpha ,f\to \beta }$ as input. When $f\left(x\right)=0$ remains constant, output the converted ciphertext $CT_{\beta }$, otherwise output ⊥;
7. ReEnc−VerVKα→β,CTα,CTβ: If the original ciphertext’s conversion to the re-encryption ciphertext is performed correctly, the output of the authentication algorithm is valid, otherwise output ⊥ (invalid ciphertext).

Correctness. In an AB-VCPRE scheme, correctness has the following two requirements:

1. Decryption correctness.

For security parameter n, attribute vectors $x={\left\{x_{i}\right\}}_{i\in \left[l\right]}$, message $\mu \in {\left\{0,1\right\}}^{m}$, the equations below hold

(5) $$Dec(\mathrm{pp},sk_{\alpha },Enc\left(pp,pk_{\alpha },\mu ,x\right))=\mu ;$$

(6) Decpp,skβ,ReEncpp,RKα,f→β,CTα=μ,

where the decryption error is negligible.

2. Verification correctness.

Verification correctness is satisfied using an AB-VCPRE scheme. We have the probability PrReEnc−Ver(VKα→β,CTα,CTβ)=1=1 if all converted ciphertexts $CT_{\beta }$ are produced by the re-encryption keys $RK_{\alpha ,f\to \beta }$ and ReEnc(pp,RKα,f→β,CTα).

### 4.2. Security Model

**Definition** **6.** *To demonstrate the CPA security of the AB-VCPRE scheme, the game between challenger* C *and adversary* A *is used.*

Init. Before seeing the public parameter pp, adversary A declares a vector of attributes $x^{*}$.

Setup. Initialize the public parameters pp in Challenger C and use the KeyGen algorithm to obtain $\left(sk_{\theta },pk_{\theta }\right)$, and transmit pp and $pk_{\theta }$ to A.

Query phase 1. A chooses some queries as the following:

- KeyGen query$O_{KeyGen}$: A performs a key query. C runs $KeyGen\left(pp,\beta \right)$ to produce the $\left(pk_{\beta },sk_{\beta }\right)$;
- ReKeyGen queryOReKeyGen: C runs ReKeyGenpp,skα,pkβ,f to provide $rk_{\alpha ,f\to \beta }$ when C receives a re-encryption key query, where $f\left(x^{*}\right)=0$ and $pk_{\beta }=KeyGen\left(pp,\beta \right)$. And C responds with verification key by running algorithm $HS.KeyGen(n,d^{hs},N)$;
- ReEnc query OReEnc: A sends $\left(CT_{\alpha },\;x,\;f\right)$ to C where $x\neq x^{*}$ and $f\left(x\right)=0$, C computes a re-encryption key $rk_{\alpha ,f\to \beta }$ as in OReKeyGen and returns a re-encrypted ciphertext $CT_{\beta }$ by running ReEncpp,RKα,f→β,CTα.

Challenge phase. A chooses two messages of the same length $\mu_{0}^{*}$ and $\mu_{1}^{*}$($\mu_{0}^{*}\neq \mu_{1}^{*}$), C executives $CT^{*}\leftarrow Enc\left(pp,pk_{\theta },x^{*},\mu_{b}^{*}\right)$, where $b\in \left\{0,1\right\}$, and gives back the original ciphertext from $CT^{*}$ to A.

Query phase 2. Similar to phase 1, A keeps asking the query.

Guess. $b^{\prime }\in \left\{0,1\right\}$ is guessed by A, and if $b=b^{\prime }$, the game winner is A.

The benefits of A are described as $\mathrm{Pr}\left[b^{\prime }=b\right]=1/2+negl\left(n\right).$

## 5. Our Scheme

### 5.1. Our Scheme Composition

Using the LWE difficulty problem as a basis and the homomorphic signature algorithm, this paper proposes an AB-VCPRE scheme.

1.
$S\mathrm{et}up\left(n\right)$

Let security parameters n∈Z, where $m\geq \left\lceil 6n\log\left(q\right)\right\rceil$, $q/4\geq B\cdot {\left(m+1\right)}^{O\left(d\right)}$.

①

Central agency generates random security parameters prime q, an error sampling algorithm χ for B-bounded distributions, $B\geq \sqrt{n}\cdot \omega \left(\log n\right)$. The boolean circuit’s maximum depth is d, the number of attributes is ℓ, and the Gaussian parameter is σ, $\sigma =\omega \left({\left(m+1\right)}^{d+1}\right)\cdot \omega \left(\sqrt{\log m}\right)\;$;

②

Create the corresponding trapdoor matrix $T_{A_{\alpha }}\in {\mathbb{Z}}_{q}^{m\times m}$ and the matrix $A_{\alpha }\in {\mathbb{Z}}_{q}^{n\times m}$ by running algorithm $TrapGen\left(1^{n},1^{m},q\right)$;

③

Select ℓ uniform matrices $B_{1},\ldots ,B_{\ell }\in {\mathbb{Z}}_{q}^{n\times m}$ with random.

④

Output public parameters $pp:=\left({\left\{B_{i}\right\}}_{i\in \left[\ell \right],\chi },\chi \right)$.

2.
$KeyGen\left(pp,\alpha \right)$

Randomly select a matrix $D_{\alpha }\in {\mathbb{Z}}_{q}^{n\times m}$, and run $R_{\alpha }\leftarrow Sample\mathrm{Pr}e\left(A_{\alpha },T_{\alpha },D_{\alpha },\sigma \right)$, such that $A_{\alpha }R_{\alpha }=-D_{\alpha }$.

Output $pk_{\alpha }=(A_{\alpha },D_{\alpha }),sk_{\alpha }=(R_{\alpha },T_{\alpha })$.

3.
$Enc(pp,pk_{\alpha },\mu ,x)$

①

Given the plaintext $\mu \in {\left\{0,1\right\}}^{m}$, attribute vectors $x\in {\left\{0,1\right\}}^{\ell }$, where $x={\left\{x_{i}\right\}}_{i\in \left[\ell \right]}$. Select random vectors $s\leftarrow {\mathbb{Z}}_{q}^{n}$, error vectors $e_{1},e_{2}\leftarrow \chi^{m}$;

②

Compute $cc=(c_{1},c_{2})$:

(7) $$c_{1}=A_{\alpha }^{T}s+e_{1},c_{2}=D_{\alpha }^{T}s+e_{2}+\left\lfloor q/2\right\rfloor \mu .;$$

③

ca should be set to ∅ if x is null or none. Or else randomly choose ℓ uniform matrices $S_{i}\leftarrow {\left\{-1,1\right\}}^{m\times m}$ at random, calculate

(8) $$ca=\left({\left\{c_{i}={\left(x_{i}G+B_{i}\right)}^{T}s+S_{i}^{T}e_{1}\right\}}_{i\in \left[\ell \right]}\right)\in {\mathbb{Z}}_{q}^{\ell m}.$$

Output ciphertext $CT_{\alpha }:=(cc,ca)$;

4.
$Dec(pp,sk_{\alpha },CT_{\alpha })$

Input $sk_{\alpha }=(R_{\alpha },T_{\alpha })$, $CT_{\alpha }=(cc,ca)$.

①

Compute $\hat{\mu }=c_{2}+R_{\alpha }^{T}c_{1}$. Set $\mu_{i}=1$ for $i\in \left[m\right]$ if $|q/2-{\hat{\mu }}_{i}|<q/4$, or else set $\mu_{i}=0$.

Output $\mu \in {\left\{0,1\right\}}^{m}$;

5.
ReKeyGen(pp,skα,pkβ,f)

Input $pk_{\beta }=(A_{\beta },D_{\beta })$, $sk_{\alpha }=(T_{\alpha },R_{\alpha })$, $pp=\left({\left\{B_{i}\right\}}_{i\in \left[\ell \right],\chi },\chi \right)$, a policy $f\in F_{\ell .d}$.

①

Randomly selected matrices $E_{1}\leftarrow \chi^{2km\times n}$, $E_{2},E_{3}\leftarrow \chi^{2km\times m}$, s is the Gaussian parameter, and $s=\omega ({\left(m+1\right)}^{d+3/2})$.

②

Let $B_{f}=Eval_{pk}(f,B_{1},\ldots ,B_{\ell })$, $F=(A_{\alpha }|B_{f})\in {\mathbb{Z}}^{n\times 2m}$. Running $T_{\alpha ,f}\leftarrow SampleBasisLeft(A_{\alpha },B_{f},T_{\alpha },s)$. Generate the basic $T_{\alpha ,f}$ for F.

③

Execute algorithm $Sample\mathrm{Pr}e(F,T_{\alpha ,f},-D_{\alpha },\sigma )$ to produce $R_{\alpha ,f}$, in order to obtain $FR_{\alpha ,f}=-D_{\alpha }$, of which $R_{\alpha ,f}\in {\mathbb{Z}}^{2m\times m}$. Compute the re-encryption key:

(9) $$Q=\left[\begin{array}{cc} E_{1}A_{\beta }+E_{2} & E_{1}D_{\beta }+E_{3}+Power2_{q}(R_{\alpha ,f})\\ 0_{m\times m} & I_{m\times m} \end{array}\right]\in {\mathbb{Z}}_{q}^{(2km+m)\times 2m};$$

④

Creating the verification key using algorithm $HS.KeyGen(n,d^{hs},N)$ and signature private key (hsvk,hssk), parse each line of Q as $w_{i}\in {\mathbb{Z}}_{q}^{2m}(1\leq i\leq 2mk+m)$, then use the signature algorithm to sign $w_{i}$ as $\sigma_{i}=HS.Sign(hssk,w_{i})$;

⑤

To validate the signature, publish hsvk. Deliver Q and the associated signature $\left\{RK_{\alpha ,f\to \beta }=Q,\sigma_{i}(1\leq i\leq 2mk+m)\right\}$ across a secure channel to the proxy server;

6.
ReEnc(pp,RKα,f→β,CTα) 

Input $pp=\left({\left\{B_{i}\right\}}_{i\in \left[\ell \right],\chi },\chi \right)$, $RK_{\alpha ,f\to \beta }=Q$, $CT_{\alpha }=(cc,ca)$.

①

Output ⊥ if f(x)≠0 or ca=∅, or else $c_{3}=Eval_{ct}(f,{\left\{\left(x_{i},B_{i},p_{i}\right)\right\}}_{i\in \left[\ell \right]})$, ${\tilde{c}}_{1,3}=$$\left(\left[c_{1};c_{3}\right]\right)$. The proxy performs the ciphertext conversion $(c_{1}^{{}^{\prime }T}|c_{2}^{{}^{\prime }T})=\left[{\tilde{c}}_{1,3}^{T}|c_{2}^{T}\right]\cdot Q$;

②

The valuation circuit is $g_{C_{\alpha }}(Q)=\left[{\tilde{c}}_{1,3}^{T}|c_{2}^{T}\right]\cdot Q$, and the evaluation algorithm from HS creates a signature $\sigma_{\alpha \to \beta }=HS.SignEval(g_{C_{\alpha }},\sigma_{i}(1\leq i\leq 2mk+m))$.

Output $CT_{\beta }=\left(cc^{\prime }=(c^{\prime }{}_{1},c^{\prime }{}_{2}),ca^{\prime }=\emptyset ,\sigma_{\alpha \to \beta }\right)$ as converted ciphertext;

7.
ReEnc−Ver(hsvk,CTα,CTβ)

Input verification key hsvk, original ciphertext $CT_{\alpha }=(cc=(c_{1},c_{2}),\sigma_{*\to \beta })$, converted ciphertext $CT_{\beta }=(cc^{\prime }=(c^{\prime }{}_{1},c^{\prime }{}_{2}),\sigma_{\alpha \to \beta })$.

Verification algorithm output $HS.Verify(hsvk,g_{C_{\alpha }},cc^{\prime },\sigma_{\alpha \to \beta })$.

**Figure 2:**
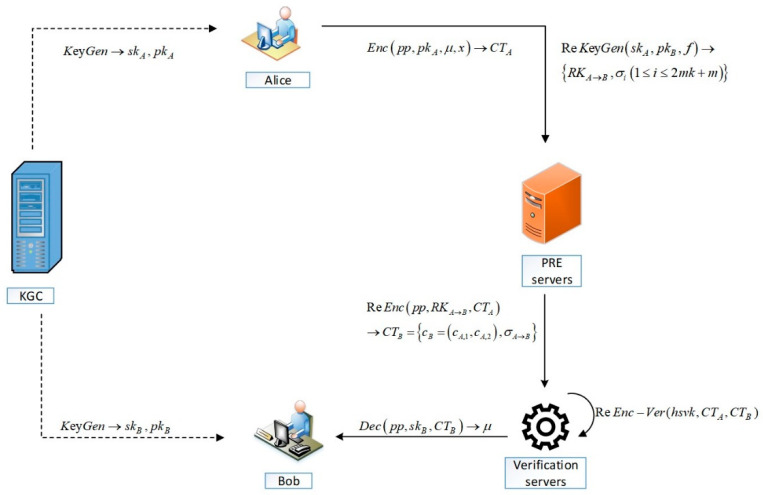
The workflow of AB-VCPRE.

Figure 2 depicts the new AB-VCPRE scheme’s workflow. If Bob wants to share Alice’s content stored on the cloud server, first KGC generates a public key and private key for Alice and Bob and sends the keys to them. Then, Alice generates the re-encryption key and original ciphertext, which are sent to the cloud server and executes the re-encryption algorithm. The cloud server delivers both the original and the re-encryption ciphertext to the authentication server after the re-encryption operation is finished. The authentication server verifies the algorithm for re-encryption. If the verification algorithm outputs 1, the authentication server sends Bob the ciphertext, Bob recovers the message by decrypting the ciphertext matching to it, otherwise output ⊥.

### 5.2. Correctness and Parameters

#### 5.2.1. The Correctness of the Original Ciphertext

With the private key $R_{\alpha }$, the original ciphertext can be decrypted.

(10) $$\begin{array}{cc} \hat{\mu } & =c_{2}+R_{\alpha }^{T}c_{1}\\ & =D_{\alpha }^{T}s+e_{2}+\left\lfloor q/2\right\rfloor \mu +R_{\alpha }^{T}\left(A_{\alpha }^{T}s+e_{1}\right)\\ & =\underset{noise}{\underset{︸}{e_{2}+e_{1}R_{\alpha }}}+\left\lfloor q/2\right\rfloor \mu \end{array}.$$

Only if the error $e_{2}+e_{1}R_{\alpha }$ does not exceed q/4 the decryption algorithm is able to correctly recover the plaintext μ. In fact, $||e_{2}+e_{1}R_{\alpha }||\leq \sqrt{m}B+m\sqrt{m}\sigma B\leq B\cdot {\left(1+m\right)}^{O\left(d\right)}$≤q/4.

#### 5.2.2. Correctness of Conversion Ciphertext

After passing one conversion, the corresponding conversion cipher is decrypted as follows:

(11) $$\begin{array}{cc} (c_{1}^{{}^{\prime }T} & \left|c_{2}^{{}^{\prime }T}\right)=\left[{\tilde{c}}_{1,3}^{T}|c_{2}^{T}\right]\cdot Q\\ \; & =\left[{\tilde{c}}_{1,3}^{T}|c_{2}^{T}\right]\cdot \left[\begin{array}{cc} E_{1}A_{\beta }+E_{2} & E_{1}D_{\beta }+E_{3}+Power2_{q}\left(R_{\alpha ,f}\right)\\ 0_{m\times m} & I_{m\times m} \end{array}\right]\\ & =\left[{\tilde{c}}_{1,3}^{T}\cdot \left(E_{1}A_{\beta }+E_{2}\right)|{\tilde{c}}_{1,3}^{T}\cdot \left(E_{1}D_{\beta }+E_{3}+Power2_{q}\left(R_{\alpha ,f}\right)\right)+c_{2}^{T}\right]\\ & =\left[{\tilde{c}}_{1,3}^{T}\cdot \left(E_{1}A_{\beta }+E_{2}\right)|{\tilde{c}}_{1,3}^{T}\cdot \left(E_{1}D_{\beta }+E_{3}\right)+{\tilde{c}}_{1,3}^{T}\cdot Power2_{q}\left(R_{\alpha ,f}\right)+D_{\alpha }s^{T}+e_{2}^{T}+\left\lfloor q/2\right\rfloor \mu^{T}\right]\\ & =\left[{\tilde{c}}_{1,3}^{T}\cdot \left(E_{1}A_{\beta }+E_{2}\right)|{\tilde{c}}_{1,3}^{T}\cdot \left(E_{1}D_{\beta }+E_{3}\right)+\left[e_{1}^{T}|e_{f}^{T}\right]R_{\alpha ,f}+e_{2}^{T}+\left\lfloor q/2\right\rfloor \mu^{T}\right] \end{array}$$

Where $A_{\beta }$ and $D_{\beta }$ are the user β‘s public keys, $||E_{1}||\leq \sqrt{2k}mB$, $||E_{2}||\leq \sqrt{2k}mB$, $||E_{3}||\leq \sqrt{2k}mB$ with overwhelming probability. By the theorem we have:

(12) $$\begin{array}{cc} {\tilde{c}}_{1,3}^{T}\cdot Power2_{q}(R_{\alpha ,f}) & ={\left[c_{1};c_{3}\right]}^{T}\cdot R_{\alpha ,f}\\ & =\left[c_{1}^{T}|c_{3}^{T}\right]\cdot R_{\alpha ,f}\\ & =\left[\left(s^{T}A+e_{1}^{T}\right)|\left(s^{T}\left(f\left(x\right)G+B_{f}\right)+e_{f}^{T}\right)\right]\cdot R_{\alpha ,f}\\ & =\left[\left(s^{T}A+e_{1}^{T}\right)|\left(s^{T}B_{f}+e_{f}^{T}\right)\right]\cdot R_{\alpha ,f}\\ & =\left[s^{T}\left[A_{\alpha }|B_{f}\right]+\left[e_{1}^{T}|e_{f}^{T}\right]\right]\cdot R_{\alpha ,f}\\ & =-s^{T}D_{\alpha }+\left[e_{1}^{T}|e_{f}^{T}\right]\cdot R_{\alpha ,f} \end{array}$$

where $R_{\alpha ,f}\leq \sqrt{2}m\sigma$, $||e_{f}||\leq B\sqrt{m}{\left(m+1\right)}^{d}$ with overwhelming probability.

The conversion ciphertext is decrypted by the private key $R_{\beta }$.

(13) $$\begin{array}{c} \left[c_{1}^{{}^{\prime }T}|c_{2}^{{}^{\prime }T}\right]\cdot \left[\begin{array}{l} R_{\beta }\\ I \end{array}\right]={\tilde{c}}_{1,3}^{T}\left(E_{1}A_{\beta }+E_{2}\right)\cdot R_{\beta }+{\tilde{c}}_{1,3}^{T}\left(E_{1}D_{\beta }+E_{3}\right)+\left[e_{1}^{T}|e_{f}^{T}\right]R_{\alpha ,f}+e_{2}^{T}+\left\lfloor q/2\right\rfloor \mu^{T}\\ =\underset{noise}{\underset{︸}{{\tilde{c}}_{1,3}^{T}E_{2}R_{\beta }+{\tilde{c}}_{1,3}^{T}E_{3}+\left[e_{1}^{T}|e_{f}^{T}\right]R_{\alpha ,f}+e_{2}^{T}}}+\left\lfloor q/2\right\rfloor \mu^{T} \end{array}$$

where:

(14) $$||{\tilde{c}}_{1,3}^{T}E_{2}R_{\beta }+{\tilde{c}}_{1,3}^{T}E_{3}+\left[e_{1}^{T}|e_{f}^{T}\right]R_{\alpha ,f}+e_{2}^{T}||\leq 2km^{2}\sqrt{m}\sigma B+2km\sqrt{m}B+2m\sqrt{m}{\left(m+1\right)}^{d}\sigma B+\sqrt{m}B\leq B{\left(m+1\right)}^{O\left(d\right)}\leq q/4$$

with overwhelming probability. Therefore, the value of μ can be decrypted correctly, i.e., the transformed ciphertext can be decrypted correctly.

In fact, the algorithm can only obtain single-hop, because in ReEnc, we set $ca^{\prime }=\emptyset$, which means that the re-encryption ciphertext cannot be encrypted again. This design is our first work and we will investigate this problem and extend it to multi-hop schemes in future work.

#### 5.2.3. Correctness of Ciphertext Verification

In the HS scheme, the re-encryption verifiability is carried out using the algorithm HS.Verify. In AB−VCPRE.ReEncpp,RKα,f→β,CTα, input the ciphertext $CT_{\alpha }$ and the re-encryption key $RK_{\alpha ,f\to \beta }$, using $g_{C_{\alpha }}(Q)=\left[Bits_{q}{\left(\left[c_{1};c_{3}\right]\right)}^{T}|c_{2}^{T}\right]\cdot Q$ as a valuation circuit, re-encryption key as circuit input, $(c_{1}^{{}^{\prime }T}|c_{2}^{{}^{\prime }T})=\left[Bits_{q}{\left(\left[c_{1};c_{3}\right]\right)}^{T}|c_{2}^{T}\right]\cdot Q$ can be seen as some computation at the message level and in $\sigma_{\alpha \to \beta }=HS.SignEval(g_{C_{\alpha }},\sigma_{i}(1\leq i\leq$2mk+m)), with signature $\sigma_{i}(1\leq i\leq 2mk+m)$ as input, and it can be interpreted as a computation of the signature level. If $\sigma_{\alpha \to \beta }$ is in fact the outcome of an honest computation based on $HS.SignEval(g_{C_{\alpha }},\sigma_{i}(1\leq i\leq 2mk+m))=\sigma_{\alpha \to \beta }$, the concept of correctness for homomorphic signature schemes holds. Then $HS.Verify(hsvk,g_{C_{\alpha }},cc^{\prime },\sigma_{\alpha \to \beta })$ can pass the verification and the verification algorithm’s accuracy is demonstrated. 

### 5.3. Security

**Theorem** **1** (Security). *The scheme we construct is CPA security under* $LWE_{n,q,\chi }$ *assumption.*

**Proof** **of** **Theorem 1.** A game-based approach is used in this proof. A challenger C can be built to resolve the LWE presumption if it is possible for an adversary A to breach the CPA’s security.

Game 0: In the original CPA attack paradigm described in Section 3, this is a true game between A and C.

Game 1: Same as game 0, but with a change in the way the common matrix ${\left\{B_{i}\right\}}_{i\in \left[\ell \right]}$ is generated. On receipt of $x^{*}$, C generates ℓ uniformly random small parametric matrices $S_{1}^{*},\ldots ,S_{\ell }^{*}\in {\left\{-1,1\right\}}^{m\times m}$, calculate $B_{i}=A^{*}S_{i}^{*}-x_{i}^{*}G$ where $i\in \left[\ell \right]$. □

**Lemma** **5.** *Game 0 is statistically indistinguishable from game 1.*

**Proof** **of** **Lemma** **5.** In game 0, ${\left\{B_{i}\right\}}_{i\in \left[\ell \right]}$ is a random uniform matrix on ${\mathbb{Z}}_{q}^{n\times m}$. In the challenge query, ${\left\{S_{i}^{*}\right\}}_{i\in \left[\ell \right]}$ is the construction of the generated challenge ciphertext $c^{*}$ random matrix. However, in game 1, $e\in \chi^{m}$ serves as the error vector and $S_{i}$ is used to generate $B_{i}$ and $c^{*}$. By Lemma 2, the distribution $\left(A^{*},{\left\{A^{*}S_{i}^{*}\right\}}_{i\in \left[\ell \right]},e\right)$ and $\left(A^{*},{\left\{A_{i}^{*}\right\}}_{i\in \left[\ell \right]},e\right)$ are statistically equivalent for any ${\left\{A_{i}^{*}\right\}}_{i\in \left[\ell \right]}\in {\mathbb{Z}}_{q}^{n\times m}$. Hence, no statistically significant difference exists between the common matrix ${\left\{B_{i}\right\}}_{i\in \left[\ell \right]}$ in games 0 and 1. This shows that there is no statistically significant difference between games 0 and 1. □

Game 2: Challenger C randomly selects $A_{\theta }$ on ${\mathbb{Z}}_{q}^{n\times m}$ with no trapdoor and utilizes the TrapGen to produce B and its trapdoor $T_{B}$.

KeyGen query $O_{KeyGen}$. A performs a key query. C run $KeyGen\left(pp,\beta \right)$ to produce the $\left(pk_{\theta },sk_{\theta }\right)$, output $pk_{\beta }$ to A.

ReKeyGen query OReKeyGen. When adversary A interrogates OReKeyGenpkα,pkβ,f to make $f\left(x^{*}\right)\neq 0$, challenger C executes $Eval_{sim}$ of Lemma 4 to create a re-encryption key.

1. $pp=\left({\left\{B_{i}\right\}}_{i\in \left[\ell \right]},\chi \right)$, $pk_{\beta }=\left(A_{\beta },D_{\beta }\right)$, policy $f\in F_{\ell ,d}$, set $B_{f}=Eval_{pk}\left(f,\left(B_{1},\ldots ,B_{\ell }\right)\right)$, a policy $F=\left(A_{\theta }|B_{f}\right)\in {\mathbb{Z}}^{n\times 2m}$;
2. Run $S_{f}^{*}\leftarrow Eval_{sim}\left(f,{\left\{\left(S_{i}^{*},x_{i}^{*}\right)\right\}}_{i\in \left[\ell \right]},A\right)$ to make $AS_{f}^{*}-f\left(x^{*}\right)G=B_{f}$. It follows from the definition of $Eval_{sim}$ that there is $||S_{f}^{*}|{|}_{2}<{\left(1+m\right)}^{d+1}$;
3. C executive $SampleBasisRight\left(A_{\theta },G,S_{f}^{*},T_{G},s\right)$ to generate short basic $T_{\theta ,f}$ of $\left(A_{\theta }|B_{f}\right)$. Run $Sample\mathrm{Pr}e\left(F,T_{\theta ,f},-D_{\theta },\sigma \right)$ to produce $R_{\theta ,f}\in {\mathbb{Z}}^{2m\times m}$, hence, an equals $FR_{\theta ,f}=-D_{\theta }$;
4. When $f\left(x^{*}\right)\neq 0$, let ${\bar{R}}_{\alpha ,f}=Power2_{q}\left(R_{\alpha ,f}\right)$, matrix $E_{1}\leftarrow \chi^{2km\times n}$, $E_{2},E_{3}\leftarrow \chi^{2km\times m}$, create the matrix

(15) $$Q=\left[\begin{array}{cc} E_{1}A_{\beta }+E_{2} & E_{1}D_{\beta }+E_{3}+Power2_{q}({\bar{R}}_{\theta ,f})\\ 0_{m\times m} & I_{m\times m} \end{array}\right]\in {\mathbb{Z}}_{q}^{(2km+m)\times 2m};$$

5. When $f\left(x^{*}\right)=0$, let ${\bar{R}}_{\alpha ,f}=Power2_{q}\left(R_{\alpha ,f}\right)$, matrix $E_{1}\leftarrow \chi^{2km\times n}$, $E_{2},E_{3}\leftarrow \chi^{2km\times m}$, select a random uniform distribution matrix $M\in {\mathbb{Z}}_{q}^{2km\times m}$, create the matrix

(16) $$Q=\left[\begin{array}{cc} E_{1}A_{\beta }+E_{2} & M+{\bar{R}}_{\theta ,f})\\ 0_{m\times m} & I_{m\times m} \end{array}\right]\in {\mathbb{Z}}_{q}^{(2km+m)\times 2m}.$$

Then A send the challenger C some re-encryption verification questions, who will then carry out the operation honestly and report the results to the adversary A.

ReEnc query OReEnc. C output ReEncpp,CTα,RKθ,f→β.

**Lemma** **6.** *Game 1 is computationally indistinguishable from game 2.*

**Proof** **of** **Lemma** **6.** The technique employed to generate the re-encryption key differs between games 1 and 2. When $f\left(x^{*}\right)=0$ hold, here is the re-encryption key:

(17) $$rk_{\theta ,f\to \beta }=\left\{\begin{array}{l} \left[\begin{array}{cc} E_{1}A_{\beta }+E_{2} & E_{1}D_{\beta }+E_{3}+{\bar{R}}_{\theta ,f}\\ 0_{m\times m} & I_{m\times m} \end{array}\right]\;in\;Game\;1\\ \left[\begin{array}{cc} E_{1}A_{\beta }+E_{2} & M+{\bar{R}}_{\theta ,f})\\ 0_{m\times m} & I_{m\times m} \end{array}\right]\;\;\;\;in\;Game\;2 \end{array}\right.$$

**Corollary** **1.** *By applying the standard mixing parameters, the ensuing distributions cannot be distinguished computationally. Otherwise, there is a useful algorithm for resolving the* $LWE_{n,q,\chi }$ *problem.*

 *1.* $\left(D,DY+F\right)$ *and* $\left(D,V\right)$*, where* $D\leftarrow {\mathbb{Z}}_{q}^{n\times m}$*,* $Y\leftarrow \chi^{m\times \ell }$*,* $F\leftarrow \chi^{n\times \ell }$*,* $V\leftarrow {\mathbb{Z}}_{q}^{n\times \ell }$*;*
 *2.* $\left(D,K,DY+F,KY+F^{\prime }\right)$ *and* $\left(D,K,DY+F,KY^{\prime }+F^{\prime }\right)$*, where* $D,K\leftarrow {\mathbb{Z}}_{q}^{n\times m}$, $Y,Y^{\prime },F,F^{\prime }\leftarrow \chi^{n\times m}$;
 *3.* $\left(D,{\left\{DY_{i}+F_{i}\right\}}_{i\in [t]}\right)$ *and* $\left(D,{\left\{V_{i}\right\}}_{i\in [t]}\right)$*, where* $D\leftarrow {\mathbb{Z}}_{q}^{n\times m}$*,* $Y_{i}\leftarrow \chi^{n\times m}$*,* $F_{i}\leftarrow \chi^{n\times \ell }$*,* $V_{i}\leftarrow {\mathbb{Z}}_{q}^{n\times \ell }$ *for* i∈[t]*,* t=poly(n).

By Corollary 1, under the LWE assumption, it is evident that game 1 and game 2 are computationally indistinguishable.

Additionally, the private key creation mechanism is undetected from game 1 to game 2, and the produced private key continues to satisfy $A_{\alpha }R_{\alpha }=D_{\alpha }$, while the re-encryption key is selected from the uniform distribution, which is similar to the standard LWE distribution. Furthermore, because homomorphic signatures are non-negligible, the adversary in the CPA game cannot offer an invalid ciphertext to pass re-encryption verification, that is, re-encryption verification provides no auxiliary capacity to the adversary.

On the other side, to demonstrate it, if A succeeds in the re-encryption verifiability game, then by interacting with challenger C, the simulator S can break the homomorphic signature’s unforgeability.

The verification key hsvk is first acquired by the simulator S from C. The re-encryption key $RK_{\theta ,f\to \beta }^{*}$ is then chosen by adversary A as the one it wants to assault, and the simulator s is provided $RK_{\theta ,f\to \beta }^{*}$ by A. To create the signature, S asks the message $RK_{\theta ,f\to \beta }^{*}$ for a homomorphic signature to obtain $\sigma_{i}\left(1\leq i\leq 2mk+m\right)$ and then gives it back to A. The challenger C then calculates $HS.Verify\left(hsvk,g_{C_{\alpha }},cc^{*},\sigma_{\theta \to \beta }^{*}\right)$ whenever A outputs a false re-encryption ciphertext $CT_{\beta }^{*}=\left(cc^{*}=\left(c_{1}^{*},c_{2}^{*}\right),ca^{*}=\emptyset ,\sigma_{\theta \to \beta }^{*}\right)$ after the simulator S has parsed it, where $g_{C_{\alpha }}$ is an evaluation circuit converted from the original ciphertext.

If A wins the verifiability of re-encryption, the forgery of A‘s signature $\sigma_{\theta \to \beta }^{*}$ can pass HS.Verify, which also counts as a valid homomorphic signature. Therefore, breaking the unforgeability of the homomorphic signature provides the same advantage as breaking the re-encryption verifiability of the AB-VCPRE scheme. When all of the aforementioned factors are considered, game 1 and game 2 are similar from the standpoint of the adversary. □

Game 3: Similar to game 2, except that the challenge cipher $CT^{*}=(c_{1}^{*},\;c_{2}^{*})\in {\mathbb{Z}}^{2m\times 1}$ given to the opponent is no longer honestly generated, but chosen evenly and randomly in ${\mathbb{Z}}^{2m\times 1}$. Due to the fact that the challenge cipher is a random factor in the cipher space, it is independent of $\mu_{0}^{*}$ and $\mu_{1}^{*}$, so there is zero advantage to the A in this game.

**Lemma** **7.** *Game 2 is statistically indistinguishable from game 3.*

**Proof** **of** **Lemma** **7.** If A distinguishes game 2 from game 3 with a non-negligible advantage, then there is a simulator S that can use the information acquired by A to resolve the $LWE_{n,q,\chi }$ problem. □

**LWE instance.** The simulator S requests the LWE prophesy device to acquire an LWE instance $\left(Y,b\right)\in {\mathbb{Z}}_{q}^{n\times 2m}\times {\mathbb{Z}}_{q}^{2m}$, possibly $\left(Y,b\right)$ is a truly random distribution or $b=Y^{T}s+e$ is a pseudo-random distribution of noise $e\in \chi^{m}$ from the LWE. 

**Public parameters.** Let $\left[A_{\theta }|D_{\theta }\right]:=Y$, sample a uniform matrix $D_{\theta }\leftarrow {\mathbb{Z}}_{q}^{n\times m}$ to generate a randomly identified public key $A_{\theta }\leftarrow {\mathbb{Z}}_{q}^{n\times m}$, select ℓ random matrices $\left(S_{1}^{*},\ldots ,S_{\ell }^{*}\right)\leftarrow {\left\{-1,1\right\}}^{m\times m}$, and let $B_{i}=A_{\theta }S_{i}^{*}-x_{i}^{*}G$ for $i\in \left[\ell \right]$. Then the common matrix $pp=({\left\{B_{i}=A_{\theta }S_{i}^{*}-x_{i}^{*}G\right\}}_{i\in [\ell ],\;\chi })$, public key $pk:=\left(A_{\theta },D_{\theta }\right)$.

**Queries.** As with game 2, B answers all of A‘s queries.

**Challenge ciphertext.** Generate challenge cipher via LWE instance

(18) $$\left[c_{1};c_{2}\right]:=z;\;$$

(19) $$\left[c_{11}^{T}|\ldots |c_{\ell \ell }^{T}\right]=c_{1}^{T}\left[S_{1}^{*}|\ldots |S_{\ell }^{*}\right].$$

The answer to A is then returned. In this case, the distribution of the challenge cipher is the same as that of game 2.

(20) $$\begin{array}{cc} z & =\left[c_{1};c_{2}\right]\\ & =Y^{T}s+e\\ & ={\left[A_{\theta }|D_{\theta }\right]}^{T}s+\left[e_{1};e_{2}\right] \end{array}$$

where $Y\leftarrow {\mathbb{Z}}_{q}^{n\times 2m}$, $s\leftarrow {\mathbb{Z}}_{q}^{n}$, $e\leftarrow \chi^{2m}$.

Challenge ciphertext:

(21) $$c_{1}=A_{\theta }^{T}s+e_{1},c_{2}=D_{\theta }^{T}s+e_{2}+\left\lfloor q/2\right\rfloor \mu ;$$

(22) $$ca={\left\{c_{i}={\left(x_{i}^{*}G+B_{i}\right)}^{T}s+{\left(S_{i}^{*}\right)}^{T}e_{1}\right\}}_{i\in \left[\ell \right]}.$$

Then through $B_{i}=A_{\theta }S_{i}^{*}-x_{i}^{*}G$, there is

(23) $$\begin{array}{cc} \left[c_{11}^{T}|\ldots |c_{\ell \ell }^{T}\right] & =\left[s^{T}A_{\theta }S_{1}^{*}+e_{1}^{T}S_{1}^{*}|\ldots \ldots |s^{T}A_{\theta }S_{\ell }^{*}+e_{1}^{T}S_{\ell }^{*}\right]\\ & =\left(s^{T}A_{\theta }+e_{1}^{T}\right)\left[S_{1}^{*}|\ldots \ldots |S_{\ell }^{*}\right]. \end{array}$$

Statistically, the challenge ciphertext is indistinguishable in the alternative scenario if Y and z are chosen consistently, according to the leftover hash lemma [25].

**Output.** The simulator S outputs A‘s guess after A predicts whether it interacts with game 2 or game 3. S can solve the $LWE_{n,q,2m,\chi }$ problem with the same probability if A can distinguish between games 2 and 3. However, the $LWE_{n,q,2m,\chi }$ problem is mysterious, so game 3 cannot be won by A.

The Proof of Theorem 1 is completed by considering game 0 to game 3.

**Theorem** **2** (Robustness). *The new AB-VCPRE scheme fulfills robustness if the homomorphic signature* $\Pi_{HS}$ *satisfies unforgeability.*

**Proof** **of** **Theorem 2.** Using a randomly selected evaluation circuit, a dishonest proxy server is able to obtain an invalid re-encryption ciphertext share and corresponding signature. However, the original ciphertext should describe the right evaluation circuit. When the correct evaluation circuit diverges from the forgery, verification fails, allowing the proxy server to convert the data truthfully.

Homomorphic signatures can be used to demonstrate the robustness of the new scheme. If A can defeat the game outlined in Definition 6, then by collaborating with C in the homomorphic signature security model, it is able to build a simulator S that compromises the homomorphic signatures’ unforgeability. Here is the procedure.

A picks the re-encryption key it wishes to attack once the simulator S receives the challenger C‘s verification key hsvk. When A sends simulator S a forged re-encryption ciphertext share $CT_{\beta }^{*}=\left(cc^{*}=\left(c_{1}^{*},c_{2}^{*}\right),ca^{*}=\emptyset ,\sigma_{\theta \to \beta }^{*}\right)$, S processes it to obtain $\left(hsvk,cc^{*}=\left(c_{1}^{*},c_{2}^{*}\right),g_{C_{\alpha }},\sigma_{\theta \to \beta }^{*}\right)$ and submits it to an oracle as a forged homomorphic signature.

If A succeeds in the robustness game, then CTβ∗≠ReEncpp,RKθ,f→β,CTθ, but $HS.Verify\left(hsvk,cc^{*}\right)=1$, this also means that $HS.Verify\left(hsvk,g_{C_{\alpha }},cc^{*},\sigma_{\theta \to \beta }^{*}\right)$ was able to pass the verification, so the simulator S successfully forged an illegal signature, which will be submitted to oracle later. This indicates that the homomorphic signature algorithm’s unforgeability has been compromised.

Thus, if the homomorphic signature algorithm $\Pi_{HS}$ meets the requirement for unforgeability, the signature is considered unforgeable. The new AB-VCPRE is capable of achieving robustness. □

**Theorem** **3** (Weak collusion resistance). *The new AB-VCPRE scheme can realize weak collusion resistance, if the LWE problem is difficult.*

**Proof** **of** **Theorem** **3.** Weak collusion resistance is that when an agent with a re-encryption key colludes with a trustee with a re-encryption key, the agent obtains only an approximate result, not an exact result.

The re-encryption key is $E_{1}A_{\beta }+E_{2}$ and $E_{1}D_{\beta }+E_{3}+Power2_{q}(R_{\alpha ,f})$, which can be further expressed as

(24) $$\left[\begin{array}{l} A_{\beta }\\ D_{\beta } \end{array}\right],E_{1}\left[\begin{array}{l} A_{\beta }\\ D_{\beta } \end{array}\right]+\left[\begin{array}{l} E_{2}\\ E_{3}+Power2_{q}\left(R_{\alpha ,f}\right) \end{array}\right]$$

This is a standard LWE distribution that is not different from unified distribution, nor can anyone obtain any useful information about private keys. After collusion, Bob encrypted the above equation with his private key $R_{\beta }$ and got $E_{2}R_{\beta }+E_{3}+Power2_{q}\left(R_{\alpha ,f}\right)$. As the noise generated during re-encryption is very low, the encryption message can be well restored by $E_{2}R_{\beta }+E_{3}+Power2_{q}\left(R_{\alpha ,f}\right)$. Therefore, in the case of collusion, the private key seems to have all been compromised. However, this is not the case. We can restore an equivalent private key, but this equivalent private key is different from the original private key. We provide the following two explanations. On the one hand, any data that can initially be decrypted by $SK_{\alpha }$ can be easily re-encrypted and read by an enemy who possesses both $RK_{\alpha ,f\to \beta }$ and $SK_{\beta }$. On the other hand, they are unable to determine the delegator’s precise private key $SK_{\alpha }$ from the equation above. Although Power2 is an easy-to-reverse feature, because it contains some noise from $E_{2}R_{\beta }+E_{3}$, you cannot obtain an exact private key from the first n-line of $E_{2}R_{\beta }+E_{3}+Power2_{q}\left(R_{\alpha ,f}\right)$. Therefore, the method proposed in this project has weak collusion resistance. □

## 6. Efficiency Analysis

Paper [15] proposed a CPRE algorithm based on DBDH, which supports fine-grained authorization and collision resistance security, however, it cannot achieve robustness. Paper [11] and paper [12] are PRE schemes with verification, both of which are robust and the method for achieving robustness is zero-knowledge proof with a decisional discrete logarithm tool, but are not as low complexity as the schemes in this paper. In addition, paper [12] is based on discrete logarithmic constructions and is not resistant to quantum attacks. Although paper [11] is a scheme using lattice construction, which seems to be resistant to quantum attacks, the robustness verification tool is a decisional discrete logarithm, so in general the scheme is not resistant to quantum attacks. Table 1 demonstrates that the approach presented in this paper is not only robust to proxy re-encryption but also simple to implement and resistant to quantum attacks.

In Table 2, the efficiency of the scheme is analyzed through plaintext space, size of ciphertext, size of re-encryption key, encryption complexity, re-encryption complexity, and robustness verification complexity. $\left|{\mathbb{Z}}_{q}\right|$ represents an integer on modulo q. $T_{p}$, $T_{e}$, $T_{s}$, $T_{v}$, and $T_{m}$ denote the computation of pairing, modular exponentiation, signature, ciphertext verification, and multiplication operation, respectively. $T_{h}$, $T_{GVP}$, respectively, represent the time spent for the hash function and the GVP algorithm. Table 2 demonstrates that the computational complexity of the literature [15] is worse than that of the proposed scheme, and is not robust. In terms of robustness verification complexity, when a boolean circuit evaluates the original signature, homomorphic signature computation is a boolean operation that is more straightforward and effective. Here, we choose the linear homomorphic signature scheme based on the difficult problem of SIS on the lattice proposed in paper [26] for comparison. Compared with the scheme [12], the proposed scheme has better re-encryption complexity, encryption complexity, and robustness verification complexity. Compared with the scheme [11], the proposed scheme in this paper only needs to pay some extra cost to encrypt the message vector, and the robustness verification complexity is lower.

## 7. Conclusions

By using homomorphic signatures, this paper proposes an AB-VCPRE scheme, which solves the problem of being unable to detect illegal proxy behavior in traditional PRE schemes. The scheme is robust enough to allow proxy servers that have sent invalid transformed ciphertext shares to be detected. In terms of security, the scheme is CPA security based on a LWE problem and is resistant to quantum attacks. In terms of efficiency, the scheme has advantages in re-encryption and robustness verification computational efficiency. In addition, there is some room for improvement in the performance of our solutions, and constructing a multi-hopping PRE scheme will be the focus of our next work.

## Figures and Tables

**Table 1:** Comparison of related work.

	Construction Tool	Resisting Quantum Attack	Robustness	Method for Robustness	Tool for Robustness
Scheme [15]	DBDH	No	No	None	None
Scheme [12]	Discrete logarithm	No	Yes	zero-knowledge proof	Decisional discrete logarithm
Scheme [11]	Lattice	No	Yes	zero-knowledge proof	Decisional discrete logarithm
Our scheme	Lattice	Yes	Yes	Homomorphic signature	Lattice

**Table 2:** Computational and communication complexity comparison.

	Message	Size of Ciphertext	Size of Re-Encryption Key	Encryption Complexity	Re-Encryption Complexity	Verification Complexity
Scheme [15]	{0,1}	8$\left|{\mathbb{Z}}_{q}\right|$	8$\left|{\mathbb{Z}}_{q}\right|$	$T_{p}+8T_{e}+T_{s}$	$2T_{p}+T_{e}+T_{v}$	None
Scheme [12]	${\left\{0,1\right\}}^{m}$	4$\left|{\mathbb{Z}}_{q}\right|$	6$\left|{\mathbb{Z}}_{q}\right|$	$3T_{e}+T_{m}$	$3T_{e}+T_{m}$	$2T_{e}+T_{h}$
Scheme [11]	{0,1}	$\left(n+1\right)\left|{\mathbb{Z}}_{q}\right|$	$\left(nm+1\right)\left(n+1\right)\left|{\mathbb{Z}}_{q}\right|$	$2T_{m}$	$2T_{m}$	$\left(nm+1\right)\left(n+1\right)T_{e}$
Our scheme	${\left\{0,1\right\}}^{m}$	$\left(\ell +2\right)m\left|{\mathbb{Z}}_{q}\right|$	$\left(4k+2\right)m^{2}\left|{\mathbb{Z}}_{q}\right|$	$5T_{m}$	$3T_{m}+T_{s}$	$T_{h}+T_{GVP}$

## Data Availability

Not applicable.
